# Elevated Carbon Dioxide Alleviates Aluminum Toxicity by Decreasing Cell Wall Hemicellulose in Rice (*Oryza sativa*)

**DOI:** 10.3389/fphys.2017.00512

**Published:** 2017-07-18

**Authors:** Xiao Fang Zhu, Xu Sheng Zhao, Bin Wang, Qi Wu, Ren Fang Shen

**Affiliations:** ^1^State Key Laboratory of Soil and Sustainable Agriculture, Institute of Soil Science, Chinese Academy of Sciences Nanjing, China; ^2^University of Chinese Academy of Sciences Beijing, China

**Keywords:** aluminum (Al) stress, cell wall, CO_2_, hemicellulose, nitric oxide (NO), root elongation

## Abstract

Carbon dioxide (CO_2_) is involved in plant growth as well as plant responses to abiotic stresses; however, it remains unclear whether CO_2_ is involved in the response of rice (*Oryza sativa*) to aluminum (Al) toxicity. In the current study, we discovered that elevated CO_2_ (600 μL·L^−1^) significantly alleviated Al-induced inhibition of root elongation that occurred in ambient CO_2_ (400 μL·L^−1^). This protective effect was accompanied by a reduced Al accumulation in root apex. Al significantly induced citrate efflux and the expression of *OsALS1*, but elevated CO_2_ had no further effect. By contrast, elevated CO_2_ significantly decreased Al-induced accumulation of hemicellulose, as well as its Al retention. As a result, the amount of Al fixed in the cell wall was reduced, indicating an alleviation of Al-induced damage to cell wall function. Furthermore, elevated CO_2_ decreased the Al-induced root nitric oxide (NO) accumulation, and the addition of the NO scavenger c-PTIO (2-(4-carboxyphenyl)-4,4,5,5-tetramethylimidazoline-1-oxyl-3-oxide) abolished this alleviation effect, indicating that NO maybe involved in the CO_2_-alleviated Al toxicity. Taken together, these results demonstrate that the alleviation of Al toxicity in rice by elevated CO_2_ is mediated by decreasing hemicellulose content and the Al fixation in the cell wall, possibly via the NO pathway.

## Introduction

Atmospheric levels of carbon dioxide (CO_2_), which is the most important gas contributing to global climate change (IPCC, 2007), have risen from 280 μL·L^−1^ during the Industrial Revolution to about 391 μL·L^−1^ today (Conway and Tans, 2011), and are predicted to peak between 490 and 1,260 μL·L^−1^ in the coming decades (Houghton et al., 2001). As a critical substance converted by plants into sugars and other carbohydrates, the increasing concentration of atmospheric CO_2_ has major influences on plant growth, such as affecting the acquisition of nutrients. Kogawara et al. (2006) demonstrated that the phosphate (P) demand of Japanese red pine (*Pinus densiflora*) was altered under different CO_2_ concentrations, and Jin et al. (2009) proposed that elevated CO_2_ improves tomato (*Solanum lycopersicum*) iron nutrition; however, whether elevated CO_2_ influences plant responses to metal toxicity such as Al is still a key unresolved issue.

Aluminum (Al), the most abundant metal on earth, inhibits root elongation in plants at very low concentrations, which in turn hinders their acquisition of nutrients (Kochian, 1995). As a result, the production and the quality of crops growing on Al-polluted land are decreased (Matsumoto, 2000). Al mainly exists as Al^3+^ in soils with a pH below 5, which is the most toxic form to plants. Extensive studies have reported that Al disrupts a set of physiological and molecular processes, such as cell wall biosynthesis and calcium metabolism, as well as distorting the structure of the plasma membrane (Panda et al., 2009); however, the primary mechanisms underlying the Al-induced inhibition of root growth remain elusive.

Plants use both internal and external mechanism to cope with Al stress (Kochian et al., 2005). To achieve external detoxification, plants either exclude Al from the root tip through secretion of Al-binding organic acids such as citrate, oxalate, and malate (Kochian et al., 2004), or bind Al in the cell wall (Delhaize et al., 1993; Zhu et al., 2013), both of which directly prevent Al from entering the cell (Magalhaes et al., 2007). The internal detoxification mechanism requires the chelation of Al inside the vacuoles (Ma, 2007). However, it is unclear how plants sense and respond to Al stress under conditions of elevated CO_2_.

Rice (*Oryza sativa*) is one of the most important crops worldwide and also is the most Al-resistant crop (Fageria, 1989), which is mediated by the expression of Al-responsive gene (Tsutsui et al., 2012), such as *ART1* (for Al resistance transcription factor 1), *STAR1* (*for sensitive to Al rhizotoxicity1*), *STAR2* and *ASR5* (*Abscisic acid, Stress, and Ripening 5*). ART1 is a C_2_H_2_-type zinc finger transcription factor that localized in the nucleus of all root cells, regulates the expression of 31 genes related to Al tolerance in rice (*Oryza sativa*; Yamaji et al., 2009), including *STAR1* and *STAR2. STAR1* encodes a nucleotide binding domain, and interacts with OsSTAR2 to form a ABC transporter that responsible for the transporation of the UDP-glucose, a substrate used to modify the cell wall and mask the Al-binding sites, while ASR5 is a key transcription factor that mediates the expression of *STAR1* through binding to the *STAR1* promoter region (Arenhart et al., 2014). Using two typical rice cultivars, “Kasalath” (Kas) and “Nipponbare” (Nip), with different sensitivities to Al stress (Yokosho et al., 2016), the current study demonstrates that elevated CO_2_ can alleviate Al toxicity through the induction of physiological and molecular changes that reduce Al accumulation in the plants.

## Materials and methods

### Plant materials and growth conditions

Rice subspecies *O. sativa* ssp. *indica* cultivar “Kasalath” and *O. sativa* ssp. *japonica* cultivar “Nipponbare” were used in the present study. Seeds were surface-sterilized using a 1% sodium hypochlorite solution prior to germination on filter paper soaked in sterilized water. After germination the seedlings were transferred to a CaCl_2_ solution (0.5 mM, pH 4.5) for 3 days. The 3-day-old seedlings were then subjected to a 24 h treatment of the same CaCl_2_ solution in either ambient CO_2_ (400 μL·L^−1^), termed “CK” (control check), or elevated CO_2_ (600 μL·L^−1^), termed “CO_2_”. Two further treatments were generated by the inclusion of 50 μM Al to the CaCl_2_ solution in either the CK conditions (termed “Al”) or to the elevated CO_2_ condition (termed “Al + CO_2_”). The rice seedlings were grown in an environmentally controlled glasshouse, with a photoperiod of 14 h light (26°C) and 10 h dark (23°C). The relative humidity was around 60%.

For experiments involving the application of the NO scavenger c-PTIO (2-(4-carboxyphenyl)-4,4,5,5-tetramethylimidazoline-1-oxyl-3-oxide), six further treatments were used. The CK treatment with additional c-PTIO (10 μM) were denoted as “c-PTIO”, while an elevated CO_2_ treatment using the CaCl_2_ solution containing 10 μM c-PTIO was also performed “CO_2_ + c-PTIO”. The Al treatments were also performed with the addition of 10 μM c-PTIO (“Al+c-PTIO”), and a final treatment combining Al + CO_2_ with 10 μM c-PTIO was termed “Al + CO_2_ + c-PTIO”. The final pH of all treatment solutions was 4.5.

### Effect of Al and lanthanum (La) on root elongation

The elongation of the root before and after various treatments (50 μM Al or 10 μM La under “CK” or “CO_2_” condition) was measured using a ruler. Relative root growth was defined as the increase in root length as a percentage of the root for a treatment compared to the increase in the CK-treated plants.

### Root apex and root cell wall extraction

The root apex of rice seedlings was cut to extract the cell wall as described by Yang et al. (2008). Briefly, the root apex (0–1 cm root tip) was ground in liquid nitrogen, after which 5 mL of 75% ethanol was added for a 20 min incubation. The homogenate was centrifuged for 8 min at 12,000 g, and the resulting pellet was washed with 5 mL acetone, then with 5 mL methanol:chloroform (1:1), and finally with 5 mL methanol. The remaining precipitate was defined as the cell wall, and was freeze-dried for further use.

### Al content measurement

The Al in the root apex and the root cell walls was extracted in 1 mL 2 M HCl with occasional shaking for 24 h, followed by centrifugation at 12,000 g for 5 min. The Al content in the supernatant was analyzed by inductively coupled plasma-atomic emission spectrometry (ICP-AES).

### Root cell wall hemicellulose and total sugar measurement

To extract the cell wall hemicellulose, the cell wall material was treated with sterilized water at 100°C three times, after which 24% KOH containing 0.1% NaBH_4_ was added to the remaining pellet twice, for 12 h each time, before centrifugation at 12,000 g for 15 min. After each KOH extraction, the supernatant was collected and combined to yield the hemicellulose.

The content of total sugars in hemicellulose was used to quantify the hemicellulose content of the cell walls. First, 200 μL of the extracted hemicellulose, 10 μL 80% phenol (v/v), and 1 mL 98% H_2_SO_4_ were incubated together at 30°C for 15 min. This solution was subsequently boiled in 100°C water bath for another 15 min, after which the solution was chilled and its absorbance of 490 nm light was determined.

### Examination of citrate efflux

Exudates from the roots of seedlings subjected to various treatments were collected, and their roots were weighed. The citrate in the root exudates was isolated and measured as described by Zhu et al. (2015).

### Measurement of root NO accumulation

The accumulation of NO in Kas roots was visualized using a NO probe, DAF-FM DA (4-amino-5-methylamino-2,7-difluorofluorescein diacetate). First, after washing with 4-(2-hydroxyethyl)-1-piperazineethanesulfonic acid (HEPES)-KOH (pH 7.4), the root apex was immersed in 200 μL DAF-FM DA (10 μM) for 30 min in dark. Excess fluorescence was removed by washing with HEPES-KOH (pH 7.4) three times, then visualized using an Eclipse 80i upright microscope with the following filters: EX 460-500, DM 505, and BA 510-560 (Nikon, Minato, Tokyo, Japan). The intensity of the fluorescence was calculated using Photoshop 7.0 (Adobe Systems Inc., San Jose, CA, USA).

### Gene expression analysis

RNA was extracted from the root apexes of the treated rice seedlings and reverse-transcribed according to Zhu et al. (2012). The total volume of the real-time PCR mixture was 10 μL, which comprised 0.2 μL each of the forward and reverse primers, 3.6 μL RNase-free water, 5 μL SYBR Premix ExTaq (Takara Bio, Inc., Kusatsu, Shiga, Japan) and 1 μL cDNA. The sequences for the gene-specific primers were as follows: *OsNRAT1* (forward: 5′-GAGGCCGTCTGCAGGAGAGG-3′; reverse: 5′-GGAAGTATCTGCAAGCAGCTCTGATGC-3′); *OsFRDL4* (forward: 5′-CGTCATCAGCACCATCCACAG-3′; reverse: 5′-TCATTTGCGAAGAAACTTCCACG-3′); *OsSTAR1* (forward: 5′-TCGCATTGGCTCGCACCCT-3′; reverse: 5′-TCGTCTTCTTCAGCCGCACGAT-3′); and *OsALS1* (forward: 5′-GGTCGTCAGTCTCTGCCTTCTC-3′; reverse: 5′-CCTCCCCATCATTTTCATTTGT-3′). Expression data were normalized with the expression level of *OsHISTONE* (forward: 5′-AGTTTGGTCGCTCTCGATTTCG-3′; reverse: 5′-TCAACAAGTTGACCACGTCACG-3′; Xia et al., 2010; Yokosho et al., 2011; Huang et al., 2012). Each cDNA sample was run in triplicate.

### Statistical analysis

Each experiment was repeated at least three times, with one set of data shown in Results. The data were analyzed using a one-way analysis of variance (ANOVA) and a Duncan's multiple range test. Different letters on the histograms indicate statistically difference at *P* < 0.05.

## Results

### Effect of elevated CO_2_ on Al-induced inhibition of root growth

**Figure 1:**
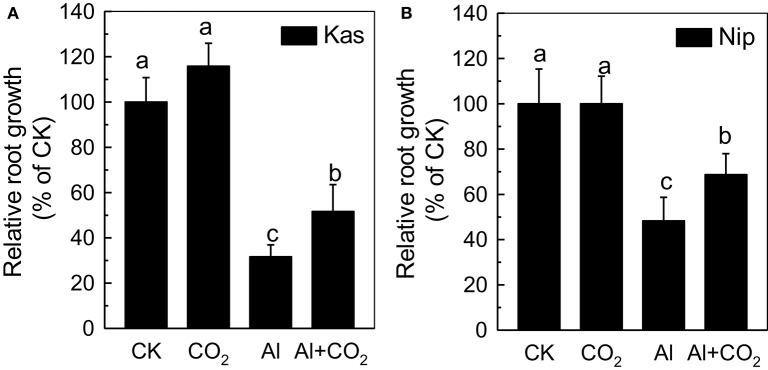
Effects of elevated CO_2_ on the relative root growth of “Kasalath” (Kas) **(A)** and “Nipponbare” (Nip) **(B)**. Three-day-old rice seedlings were treated with 0.5 mM CaCl_2_ solution with or without 50 μM Al under ambient (400 μL·L^−1^; CK) or elevated (600 μL·L^−1^; CO_2_) CO_2_ for 24 h (pH 4.5). Root length was measured before and after treatment. Data are means ± *SD* (*n* = 10). Columns with different letters are significantly different at *P* < 0.05.

Three-day-old Kas and Nip rice plants grown for a further 24 h in ambient CO_2_ (CK) in a medium containing 50 μM Al showed a significant reduction in root length relative to CK, producing roots 69 and 52% shorter than the control, respectively (Figure 1; Supplemental Table 1). This inhibition of root elongation was significantly less severe in plants grown in elevated CO_2_, with reductions of only 49% in Kas and 32% in Nip, indicating that elevated CO_2_ can alleviate Al toxicity in rice. Furthermore, the elongation of the Kas and Nip roots in control conditions (without Al) showed almost no difference between ambient and elevated CO_2_ treatments (Figure 1; Supplemental Table 1), showing that elevated CO_2_ did not affect root growth directly. However, elevated CO_2_ did not affect the root growth under other trivalent metals stress such as La (Supplemental Figure 1), indicating that elevated CO_2_ only can alleviate Al toxicity. Furthermore, as elevated CO_2_ had a similar effect on the root elongation of Nip and Kas in the presence of Al, and Kas is the more Al-sensitive cultivar (Figure 1; Yokosho et al., 2016), thus all of the following experiments were performed in Kas.

### Effect of elevated CO_2_ on rice root apex Al content

**Figure 2:**
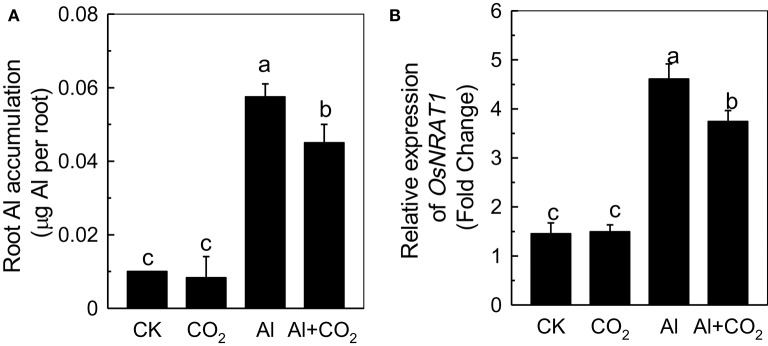
Effects of elevated CO_2_ on Al accumulation **(A)** and the expression of *OsNRAT1* **(B)** in Kas roots. Three-day-old rice seedlings were treated with 0.5 mM CaCl_2_ solution with or without 50 μM Al under ambient (400 μL·L^−1^; CK) or elevated (600 μL·L^−1^; CO_2_) CO_2_ for 24 h (pH 4.5). The root apex was excised for Al content measurement **(A)** and for RNA extraction **(B)**. Expression levels of plants grown in ambient CO_2_ without Al treatment were assigned as expression level of 1. Data are means ± *SD* (*n* = 4). Columns with different letters are significantly different at *P* < 0.05.

To uncover the potential mechanism behind the alleviation of Al toxicity by elevated CO_2_, Al accumulation in root apex was determined. There was ~0.01 μg Al in the root apex in the absence of Al, which may be due to the impurity of the chemicals used (Figure 2A). Under Al stress, elevated CO_2_ significantly decreased the root apex Al content when compared with the ambient CO_2_ treatment, indicating that the alleviation of Al-induced root growth inhibition by elevated CO_2_ might achieved by reducing Al accumulation. This conclusion was further confirmed by the reduced expression level of *NRAMP ALUMINUM TRANSPORTER 1* (*NRAT1*) in the Al-stressed plants in elevated CO_2_ compared to those in ambient CO_2_ (Figure 2B), as NRAT1 is a plasma membrane-localized protein responsible for the uptake of the trivalent Al ion (Xia et al., 2010).

### Effect of elevated CO_2_ on citrate secretion

**Figure 3:**
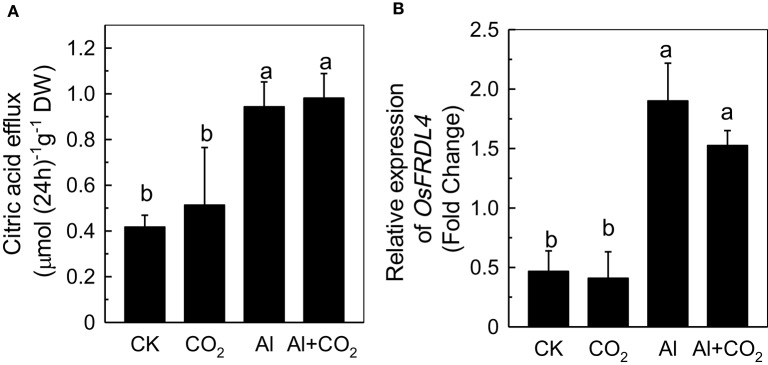
Effects of elevated CO_2_ on citric acid efflux **(A)** and the expression of *OsFRDL4* **(B)** in Kas roots. Three-day-old rice seedlings were treated with 0.5 mM CaCl_2_ solution with or without 50 μM Al under ambient (400 μL·L^−1^; CK) or elevated (600 μL·L^−1^; CO_2_) CO_2_ for 24 h (pH 4.5). Root exudates were collected **(A)** and the root apex was detached for RNA extraction **(B)**. Expression levels of plants grown in ambient CO_2_ without Al treatment were assigned as expression level of 1. Data are means ± *SD* (*n* = 4). Columns with different letters are significantly different at *P* < 0.05.

The amount of citrate in the root exudate was quantified to determine whether the reduced Al accumulation in roots under elevated CO_2_ was due to an alteration of the Al-induced secretion of citrate (Yang et al., 2008). Although Al significantly induced the secretion of citrate (Figure 3A), there was no significant difference between the ambient CO_2_ and elevated CO_2_ treatments, indicating that the differences in root Al accumulation in the two conditions could not be attributed to the secretion of citrate. This conclusion was further confirmed by the similar levels of *FERRIC REDUCTASE DEFECTIVE LIKE4* (*FRDL4*) expression between plants in the ambient and elevated CO_2_ conditions (Figure 3B), which is known to be correlated well with the secretion of citrate (Yokosho et al., 2011, 2016).

### Effect of elevated CO_2_ on root cell wall Al content and hemicellulose content

**Figure 4:**
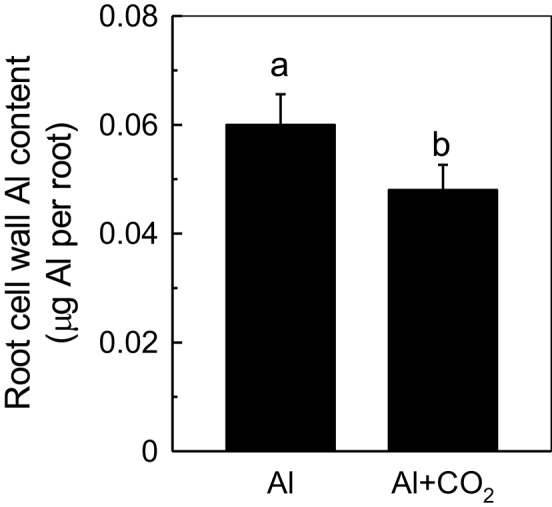
Effects of elevated CO_2_ treatment on root cell wall Al accumulation in Kas. Three-day-old rice seedlings were treated with 0.5 mM CaCl_2_ solution with or without 50 μM Al under ambient (400 μL·L^−1^; CK) or elevated (600 μL·L^−1^; CO_2_) CO_2_ for 24 h (pH 4.5). The root apex was excised for cell wall extraction. Data are means ± *SD* (*n* = 4). Columns with different letters are significantly different at *P* < 0.05.

**Figure 5:**
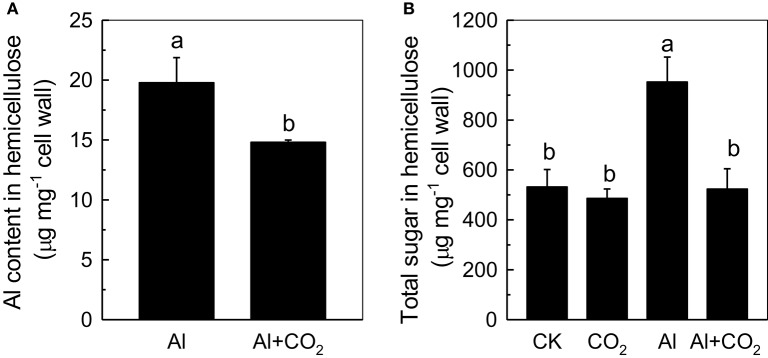
Effects of elevated CO_2_ treatment on the Al content **(A)** and the total sugar content **(B)** of Kas root cell wall hemicellulose. Three-day-old rice seedlings were treated with 0.5 mM CaCl_2_ solution with or without 50 μM Al under ambient (400 μL·L^−1^; CK) or elevated (600 μL·L^−1^; CO_2_) CO_2_ for 24 h (pH 4.5). Data are means ± *SD* (*n* = 4). Columns with different letters are significantly different at *P* < 0.05.

Recently, more and more evidences indicate that cell wall plays pivotal roles when plant in response to Al toxicity (Horst et al., 2010; Zhu et al., 2013), thus we determined the influence of elevated CO_2_ on cell wall Al accumulation in Kas root. As shown in Figure 4, less Al was accumulated in the root cell walls of Al-treated plants grown in elevated CO_2_ than those in ambient conditions (Figure 4). One of the component cell wall polysaccharides, hemicellulose, was previously shown to significantly contribute to the Al-binding capacity of the cell wall (Yang et al., 2011); therefore, its involvement in root cell wall Al accumulation was investigated. As expected, the cell walls of Al-treated plants contained significantly more hemicellulose (indicated by the total sugar content in hemicellulose) than the controls; however, when Al-treated plants were grown in elevated CO_2_, their cell walls contained significantly less hemicellulose than those grown in ambient CO_2_ (Figure 5B). This correlated well with the reduced Al accumulation in the hemicellulose of the cell walls in plants grown in elevated CO_2_ (Figure 5A), as the content of hemicellulose correlated well with the Al retention in cell wall and hemicellulose (Zhu et al., 2012), and indicated that less Al entered into the cells under elevated CO_2_, making the plants more Al resistant. It is noteworthy that Al largely accumulates in the hemicellulose of the cell wall, although a 2-fold difference in hemicellulose content in the Al vs. Al + CO_2_ treatments was found, there's only a 25% change in Al accumulation in the wall. One possible explanation is that there may be a threshold of the hemicellulose to bind Al, thus a 2-fold increment of hemicelulose under Al treatment does not lead to a 2-fold Al accumulation here, which needs further study.

### Effect of elevated CO_2_ on the expression of *OsALS1* in roots

**Figure 6:**
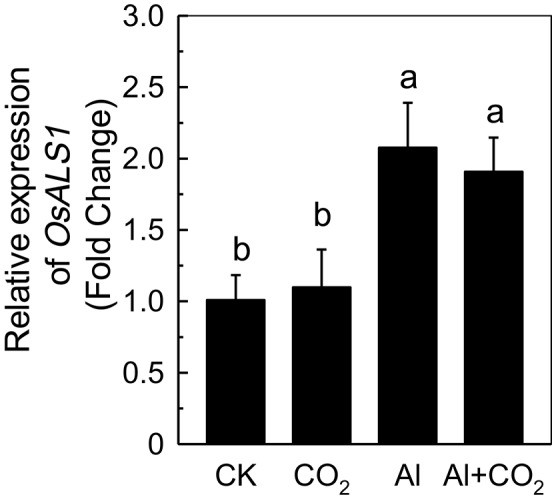
Effects of elevated CO_2_ treatment on the expression of *OsALS1* in Kas. Three-day-old rice seedlings were treated with 0.5 mM CaCl_2_ solution with or without 50 μM Al under ambient (400 μL·L^−1^; CK) or elevated (600 μL·L^−1^; CO_2_) CO_2_ for 24 h (pH 4.5). The root apex was excised for RNA extraction. Expression levels of plants grown in ambient CO_2_ were assigned as expression level of 1. Data are means ± *SD* (*n* = 4). Columns with different letters are significantly different at *P* < 0.05.

Although, most Al is fixed in the root cell wall (Ma et al., 2004), a fraction of the metal can rapidly enter into the cells, initiating an internal detoxification mechanism. OsALS1, a tonoplast transporter localized in rice root tips, can reallocate Al from the cytoplasm to the vacuole (Huang et al., 2012), and is thus essential for the internal detoxification of Al. While the expression of *OsALS1* was significantly increased in plants treated with Al, there was almost no difference in its expression between plants grown in ambient or elevated CO_2_ (Figure 6), suggesting that the *OsALS1*-based internal detoxification mechanism is not involved in the elevated CO_2_-enhanced Al resistance.

### Possible involvement of NO in the alleviation of Al toxicity by elevated CO_2_

**Figure 7:**
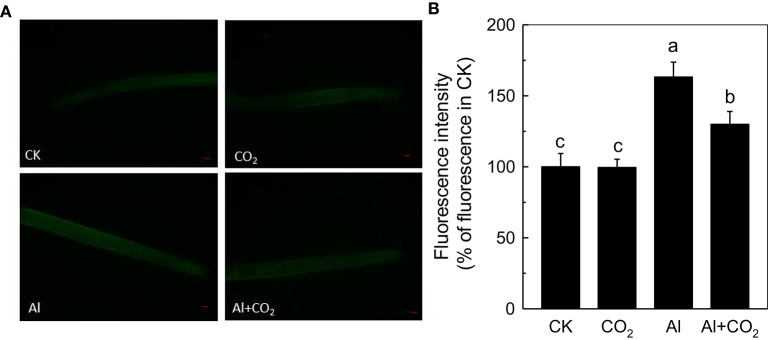
Effects of elevated CO_2_ treatment on the NO production in Kas. Three-day-old rice seedlings were treated with 0.5 mM CaCl_2_ solution with or without 50 μM Al under ambient (400 μL·L^−1^; CK) or elevated (600 μL·L^−1^; CO_2_) CO_2_ for 24 h (pH 4.5). **(A)** Photographs of NO production shown as green fluorescence in representative roots and **(B)** NO production expressed as relative fluorescence intensity (% of control). Data are means ± *SD* (*n* = 10). Scale bar = 1 mm. Columns with different letters are significantly different at *P* < 0.05.

**Figure 8:**
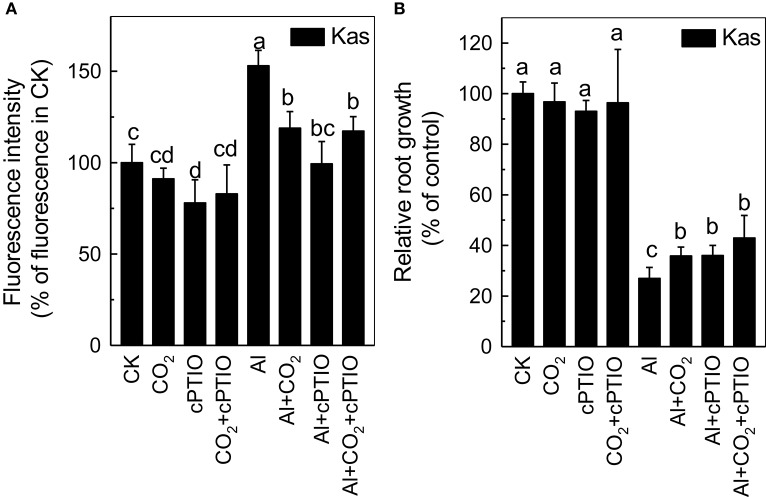
Role of NO in the elevated CO_2_alleviated Al-toxicity in Kas. Three-day-old rice seedlings were treated with a 0.5 mM CaCl_2_ solution with or without 50 μM Al under ambient or elevated CO_2_ for 24 h in the presence or absence of c-PTIO for the NO production analysis **(A)** and root length measurement **(B)**. NO production was expressed as relative fluorescence intensity (% of control). Root length was measured before and after treatment. Data are means ± *SD* (*n* = 10). Columns with different letters are significantly different at *P* < 0.05.

As elevated CO_2_ can influence endogenous NO content under different stress, such as Fe deficiency in tomato (Jin et al., 2009) and P deficiency in Arabidopsis (Niu et al., 2013), thus we then examined root NO level. Although Al significantly induced the accumulation of NO in the rice roots, the elevated CO_2_ treatment significantly reduced their NO content (Figure 7). To determine whether this reduction of NO content was involved in the enhancement of Al resistance by elevated CO_2_, a NO scavenger c-PTIO was added. The c-PTIO treatment had no influence on root growth in the absence of Al, however, Al-treated plants grown in elevated CO_2_ (Al + CO_2_ + c-PTIO) condition had a similar level of root growth as those treated with c-PTIO in ambient CO_2_ condition (Al + c-PTIO; Figure 8B; Supplemental Table 2), indicating that alleviation of Al toxicity by CO_2_ depends on NO accumulation as eliminating NO abolishes this beneficial effect (Figure 8).

## Discussion

Increases in atmospheric CO_2_ have previously been shown to stimulate plant growth, increasing the demands for macronutrients such as phosphorus and nitrogen (Lagomarsino et al., 2008), and enhancing the reutilization efficiency of iron and phosphorus where these nutrients are limited (Jin et al., 2009; Niu et al., 2013). Until now, the interaction of elevated CO_2_ with Al toxicity remained elusive. In this study, we found that elevated CO_2_ alleviated the Al-induced inhibition of root growth in rice (Figure 1) and demonstrated that elevated CO_2_ decreased the hemicellulose content of the cell wall and the amount of Al bound to hemicellulose (Figure 5). This in turn decreased the cell wall Al content (Figure 4), resulting in a more Al-resistant rice (Figure 1). Neither the external exclusion mechanism involving the secretion of the organic acids (Figure 3) nor the internal detoxification mechanism regulated by *OsALS1* (Figure 6) were found to be involved in this elevated CO_2_ alleviation of Al toxicity, but NO signaling does appear to participate in this process (Figures 7, 8). To our knowledge, this is the first study to show that elevated CO_2_ can affect Al sensitivity in rice through the modification of the Al-binding capacity of root cell wall hemicellulose.

The reduced inhibition of Al-treated plant root elongation under elevated CO_2_ is likely to be related to the reduced Al accumulation (Figures 1, 2A), as root Al levels have been shown to correlate well with Al toxicity in several studies (Frantzios et al., 2001; Wang et al., 2010; Zhu et al., 2012). OsNRAT1, part of the NRAMP (natural resistance-associated macrophage protein) family, is known to be involved in root Al accumulation in rice, as the *nrat1* loss-of-function mutant had a reduced root Al content (Xia et al., 2010). In the present study, the expression of *OsNRAT1* was up-regulated by Al (Figure 2B), as previously shown by Xia et al. (2010). We found that the *OsNRAT1* expression decreased in the elevated CO_2_ treatment, which might be responsible for the lower root Al accumulation in Al-treated plants grown in elevated rather than ambient CO_2_ (Figure 1B).

The secretion of a variety of organic acids from the root apex has been demonstrated to play an essential role in preventing Al from entering the root (Kochian et al., 2015), with citrate in soybean (Shen et al., 2005), malate in wheat (Sasaki et al., 2004), and oxalate in buckwheat (Zheng et al., 1998), all being shown to reduce Al toxicity to plants. In the present study, Al significantly induced the secretion of citrate (Figure 3A) and up-regulated the expression of *OsFRDL4* (Figure 3B), which is able to transport citrate and correlates well with the secretion of citrate in rice (Yokosho et al., 2011, 2016). Elevated CO_2_ had no further effect on the secretion of citrate in Al-treated plants (Figure 3A), indicating that citrate efflux does not contribute to the CO_2_-mediated reduction of Al accumulation in rice roots.

A large proportion of the accumulated Al in the roots is bound to the cell wall, thus, restricting Al to the cell wall is another class of exclusion-based Al-resistance mechanisms in rice. Bound Al is detrimental to the function of the cell wall, making it more rigid and decreasing the wall viscosity and elasticity, which in turn inhibits root elongation (Ma et al., 2004). Studies in rice (Yang et al., 2008) and maize (Eticha et al., 2005) have shown that the more Al that binds to the cell wall, the more sensitive the plant is to Al. Moreover, we recently identified two Arabidopsis mutants, *xth31* and *xth15*, that accumulated less Al in their root cell walls and thus were highly Al resistant (Zhu et al., 2012, 2013). Here, we found that elevated CO_2_ significantly reduced Al retention in the cell wall (Figure 4), which in turn increased Al resistance in rice (Figure 1A), indicating the operation of the cell-wall-based Al exclusion mechanism. However, the expression of *OsSTAR1* is not further up-regulated by elevated CO_2_ under Al stress (Supplemental Figure 2), indicating that the *OsSTAR1* is not involved in CO_2_-alleviated Al toxicity.

Moreover, as hemicellulose is regarded as the major site for Al binding in the cell wall (Yang et al., 2011), we investigated whether hemicellulose contributed to the reduced cell wall Al accumulation. As expected, elevated CO_2_ significantly reduced hemicellulose content and Al retention within this polysaccharide (Figure 5), indicating that hemicellulose contributes to the decreased Al accumulation in rice root cell walls under elevated CO_2_. However, the reason why elevated CO_2_ significantly reduced the production of the hemicellulose is not clear, we speculated that this may be attributed to the reduced NO accumulation, which has yet to be clarified.

Once Al enters the root cell, an internal detoxification mechanism is activated to compartmentalize Al into the vacuole; for example, Al was sequestered into the vacuole in its Al-organic acids form in hydrangea cell saps (Ma et al., 1997), and in its Al-oxalate form in the protoplasts of buckwheat leaves (Shen et al., 2002). Later, Larsen et al. (2005, 2007) identified ALS3 and ALS1, both of which can sequester Al from more sensitive organs to less sensitive tissues in Arabidopsis, with each knockout mutant exhibiting hypersensitivity to Al. Zhu et al. (2013) demonstrated that auxin negatively regulates Al tolerance by altering *AtALS1* expression, which made the growing root suffer Al toxicity. Recently, OsALS1 was characterized, and found to be localized in the tonoplast and responsible for compartmentalizing Al into the vacuoles (Huang et al., 2012). *OsALS1* is expressed in all root cells, and the knockout of *OsALS1* results in an extremely Al-sensitive plant. In the present study, we tested the expression of *OsALS1* in Al-treated plants growing in either ambient or elevated CO_2_, however, they showed similar levels of expression (Figure 6), suggesting this internal detoxifying mechanism is not involved in CO_2_-alleviated Al toxicity.

Then, how does the elevated CO_2_ decrease cell wall Al accumulation and alleviate Al toxicity? NO has previously been shown to be a crucial signaling molecule involved in Al toxicity; for instance, exacerbating Al toxicity in the rice bean (Zhou et al., 2012). In the current study, elevated CO_2_ significantly decreased the Al-induced production of NO in the roots (Figure 7), and the addition of the NO scavenger c-PTIO made no difference to the Al tolerance of plants in ambient or elevated CO2 conditions (Figure 8). This suggests that the protective effect of elevated CO_2_ to plants under Al stress may be related to the decreased accumulation NO. Interestingly, elevated CO_2_ had no effect on the root NO level when in the absence of Al (Figure 7), which may explain why elevated CO_2_ only specifically improved root elongation in plants faced with Al toxicity. Furthermore, a question is raised as to why CO_2_ elevation decreases NO levels in roots. Jin et al. (2009) reported that elevated CO_2_ can induce NO accumulation under Fe-deficient condition in tomato (*Solanum lycopersicum*), and they speculated that the elevated CO_2_ first increased the auxin levels (Teng et al., 2006), which subsequently induced the NO accumulation (Du et al., 2008). However, in the present study, CO_2_ elevation decreased NO accumulation under Al stress, and this inconsistency may be attributed to different plant cultivars, different culture condition, and etc, which needs further investigation.

In conclusion, we have demonstrated for the first time that elevated CO_2_ can alleviate Al toxicity in rice. This protection is mediated by a cell wall-based Al-exclusion mechanism involving a decrease in hemicellulose content and a reduction in the Al fixation of the hemicellulose. NO may act downstream of CO_2_ to control these processes; however, this putative mechanism requires further investigation.

## Author contributions

XFZ and RFS designed the research, XFZ, XSZ, BW, and QW performed research, XSZ and BW analyzed data. XFZ and RFS wrote the article.

### Conflict of interest statement

The authors declare that the research was conducted in the absence of any commercial or financial relationships that could be construed as a potential conflict of interest.
