# Supplementary material for: Elevated Carbon Dioxide Alleviates Aluminum Toxicity by Decreasing Cell Wall Hemicellulose in Rice (Oryza sativa)
Source: Front Physiol. 2017 Jul 18;8:512. doi: 10.3389/fphys.2017.00512 (PMC5513963; doi:10.3389/fphys.2017.00512)
Supplement: Supplementary file 1 [file DataSheet1.pdf]

Supplemental Table 1. The raw data for root lengths for Figure 1. Three-day-old rice seedlings were treated with a 0.5 mM $CaCl_2$ solution with or without 50 µM Al under ambient or elevated CO_2 for 24 h (pH 4.5). Root length was measured before and after treatment.

Before treatment	kas CK	kas CO_2	kas Al	kas Al+CO_2		Nip CK	Nip CO_2	Nip Al	Nip Al+CO_2
	3.5	3	2.1	2		2	3.3	1.7	2.2
	3	3	2.3	2		2.3	2.5	3.3	1.4
	3.4	2.8	2.6	2.5		2.5	2.6	3.2	2.5
	2.5	2.9	2.4	1.8		2	1.9	2	2.7
	3.5	3.2	4.1	5.5		2.7	1.5	2.5	2.8
	3.2	2.8	3.2	3.2		1.3	2	3.2	2.5
	3.8	2.8	2.8	2.6		2.7	1.5	3.1	1.9
	2.9	2.6	3.5	2.6		3.6	2.7	1.9	3.2
	3.5	2.6	2.5	2.9		3.5	2.5	2.2	2.5
	2.5	2.5	2.1	3		1.8	2.4	1.2	1.6
After treatment	kas CK	kas CO_2	kas Al	kas Al+CO_2		Nip CK	Nip CO_2	Nip Al	Nip Al+CO_2
	5.8	5.5	2.8	3		3.5	4.9	2.4	3
	4.7	5.4	3.1	3.8		3.4	4.2	4	2.3
	5.6	5.5	3.4	3.5		3.8	3.8	3.8	3.7
	4.8	5.4	3.1	2.7		3.3	3.3	2.6	3.6
	5.3	6	4.9	6.6		4	3	3.3	3.7
	5.3	5	3.7	4.2		2.7	3.7	3.8	3.6
	6.1	5.6	3.5	3.6		4.5	2.8	3.6	3.1
	5	5	4.1	3.6		5.2	4	2.6	4.2
	5.9	5	3	4		5.2	4.1	3.1	3.5
	4.8	4.7	2.8	4.2		3.5	4	2.2	2.7
Root elongation	kas CK	kas CO_2	kas Al	kas Al+CO_2		Nip CK	Nip CO_2	Nip Al	Nip Al+CO_2
	2.3	2.5	0.7	1		1.5	1.6	0.7	0.8
	1.7	2.4	0.8	1.8		1.1	1.7	0.7	0.9
	2.2	2.7	0.8	1		1.3	1.2	0.6	1.2
	2.3	2.5	0.7	0.9		1.3	1.4	0.6	0.9
	1.8	2.8	0.8	1.1		1.3	1.5	0.8	0.9
	2.1	2.2	0.5	1		1.4	1.7	0.6	1.1
	2.3	2.8	0.7	1		1.8	1.3	0.5	1.2
	2.1	2.4	0.6	1		1.6	1.3	0.7	1
	2.4	2.4	0.5	1.1		1.7	1.6	0.9	1
	2.3	2.2	0.7	1.2		1.7	1.6	1	1.1

Supplemental Table 2. The raw data for root lengths for Figure 8. Three-day-old rice seedlings were treated with a 0.5 mM $CaCl_2$ solution with or without 50 µM Al under ambient or elevated CO_2 for 24 h in the presence or absence of c-PTIO (pH 4.5). Root length was measured before and after treatment.

Before treatment	Kas CK	Kas CO_2	Kas cPTIO	Kas CO_2+cPTIO	Kas Al	Kas Al+CO_2	Kas Al+cPTIO	Kas Al+CO_2+cPTIO
	5.4	4.1	2.6	4.1	2.8	4	2.4	1.8
	3.5	2.7	6.5	4.5	2.8	2.9	4	2.8
	6.4	5.1	3.6	4.4	2.1	2.1	3.1	3.4
	3	3.6	4.8	4.6	3.1	3.6	4.4	3.7
	3	2.1	2.7	1.7	1.7	2.9	4	3.5
	3	3.2	3.8	4	3	1.4	1.6	4.4
	3.8	3	2.4	2.6	2.5	1.9	2	2.6
	2.8	5	2.6	3.1	2.1	4.1	2.6	2.4
	4.5	4.8	2.2	2.1	1.7	2	2.4	5.3
	4	4.5	2.2	2.4	1.5	2.4	2	2.2
After treatment	Kas CK	Kas CO_2	Kas cPTIO	Kas CO_2+cPTIO	Kas Al	Kas Al+CO_2	Kas Al+cPTIO	Kas Al+CO_2+cPTIO
	8.4	5.4	5.1	7.2	3.8	5.7	3.4	3.2
	6.3	5.4	9	7.4	3.5	3.8	4.9	3.8
	8.9	8.4	6.2	5.8	3	3.2	4	4.5
	5.8	6.4	7.2	7.7	3.8	4.7	4.8	5.1
	4.8	4.3	5.2	4.4	2.1	3.8	4.9	4.9
	5.8	6	6.5	5.8	3.6	2.4	2.4	5.2
	6.6	5.5	5	5.5	3.2	2.8	2.8	3.5
	5.4	7.5	5.1	5.5	2.8	5	3.7	3.4
	7.1	7.4	4.6	4.8	2.6	2.9	3.2	6
	6.6	7.2	4.5	5.3	2.1	3.2	2.9	3.2
Root elongation	Kas CK	Kas CO_2	Kas cPTIO	Kas CO_2+cPTIO	Kas Al	Kas Al+CO_2	Kas Al+cPTIO	Kas Al+CO_2+cPTIO
	3	1.3	2.5	3.1	1	1.7	1	1.4
	2.8	2.7	2.5	2.9	0.7	0.9	0.9	1
	2.5	3.3	2.6	1.4	0.9	1.1	0.9	1.1
	2.8	2.8	2.4	3.1	0.7	1.1	0.4	1.4
	1.8	2.2	2.5	2.7	0.4	0.9	0.9	1.4
	2.8	2.8	2.7	1.8	0.6	1	0.8	0.8
	2.8	2.5	2.6	2.9	0.7	0.9	0.8	0.9
	2.6	2.5	2.5	2.4	0.7	0.9	1.1	1
	2.6	2.6	2.4	2.7	0.9	0.9	0.8	0.7
	2.6	2.7	2.3	2.9	0.6	0.8	0.9	1

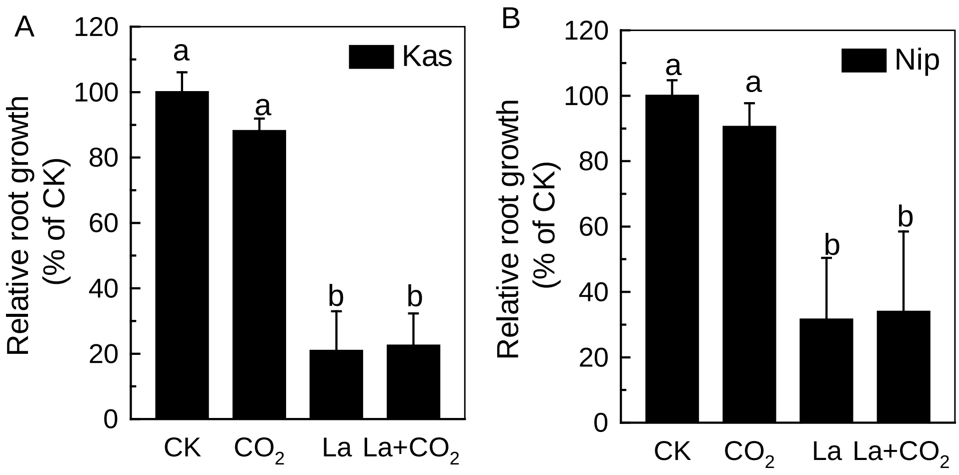

Supplemental Figure 1. Effects of elevated CO_2 on the relative root growth of 'Kasalath' (Kas) (A) and 'Nipponbare' (Nip) (B). Three-day-old rice seedlings were treated with 0.5 mM $CaCl_2$ solution with or without 10 µM La under ambient (400 $\mu L \cdot L^{-1}$; CK) or elevated (600 $\mu L \cdot L^{-1}$; CO_2) CO_2 for 24 h. Root length was measured before and after treatment. Data are means ± SD (n = 10). Columns with different letters are significantly different at $P < 0.05$.

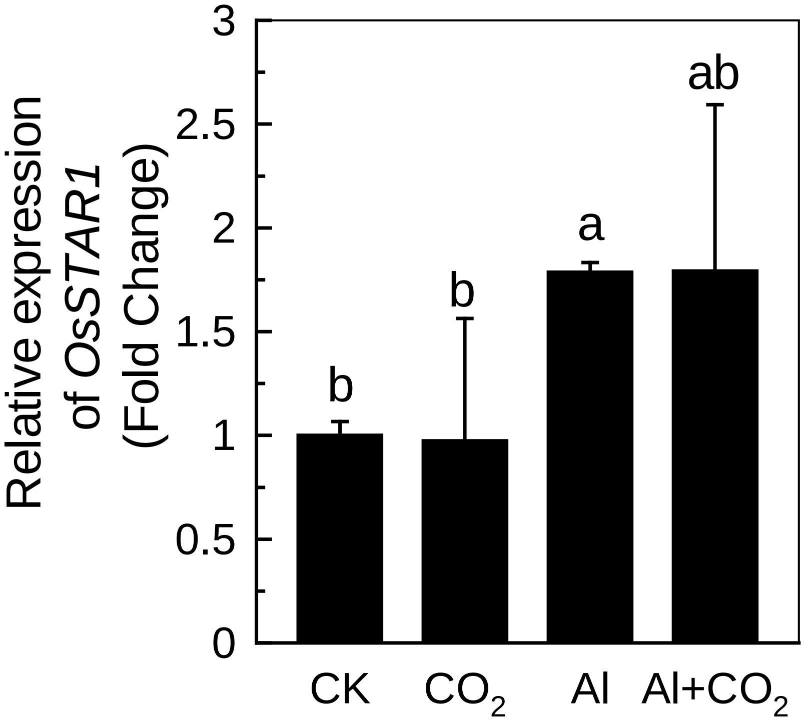

Supplemental Figure 2. Effects of elevated CO_2 treatment on the expression of *OsSTAR1* in Kas. Three-day-old rice seedlings were treated with 0.5 mM $CaCl_2$ solution with or without 50 µM Al under ambient (400 $\mu L \cdot L^{-1}$; CK) or elevated (600 $\mu L \cdot L^{-1}$; CO_2) CO_2 for 24 h. The root apex was excised for RNA extraction. Expression levels of plants grown in ambient CO_2 were assigned an expression level of 1. Data are means ± SD (n = 4). Columns with different letters are significantly different at $P < 0.05$.
